# Association between hypoglycemia and dementia in patients with diabetes: a systematic review and meta-analysis of 1.4 million patients

**DOI:** 10.1186/s13098-022-00799-9

**Published:** 2022-02-14

**Authors:** Lifen Huang, Manlian Zhu, Jie Ji

**Affiliations:** 1 Department of Geriatrics, Lishui Second People’s Hospital, Lishui, China; 2 Department of Rehabilitation, Lishui Second People’s Hospital, Fifth floor, Rehabilitation Building, 69 Huan North Road, Lishui, China

**Keywords:** Diabetes, Dementia, Hypoglycemia, Complication

## Abstract

**Background:**

Diabetes mellitus (DM) is known to be a risk factor for dementia. However, it is unclear if hypoglycemic events play a role in the risk of dementia. We aimed to systematically review evidence on the risk of dementia in DM patients based on prior hypoglycemic events.

**Methods:**

PubMed, Embase, ScienceDirect, CENTRAL, and Google Scholar databases were searched till 15th November 2021 for cohort studies assessing the risk of dementia based on prior hypoglycemic events in DM patients. Adjusted data were pooled in a random-effects model.

**Results:**

Ten studies with a total of 1,407,643 patients were included. Pooled analysis of all ten studies indicated that hypoglycemic episodes were associated with a statistically significant increase in the risk of dementia in DM patients as compared to those not experiencing hypoglycemic episodes (HR: 1.44 95% CI: 1.26, 1.65 I^2 ^= 89% p < 0.00001). The results did not change on the exclusion of any study. Sub-group analysis based on the study population, type of study, adjustment for glycated hemoglobin, gender, and the number of hypoglycemic episodes also presented similar results.

**Conclusions:**

Evidence from observational studies with a large sample size indicates that DM patients with hypoglycemic episodes are at increased risk of dementia. Anti-hyperglycemic drugs should be adequately tailored in these patients to avoid the risk of dementia.

## Background

Diabetes mellitus (DM) has been regarded as a global epidemic affecting a large number of patients worldwide. Research indicates that the incidence of DM is on the rise and around 592 million people will be affected by the disease in 2035 [1]. Regardless of the progress in therapeutics and management of DM, diabetes-related complications continue to be a major healthcare problem [2]. In addition to well-recognized complications like retinopathy, neuropathy, diabetic kidney disease, and cardiovascular disorders, DM is now a well-established risk factor for dementia [3]. Research suggests DM patients have a 25-91% increased risk of dementia as compared to non-diabetics [4]. In this context, there has been increased interest in the effect of glucose-lowering therapies and glycemic control to prevent cognitive decline [5]. While studies have indicated that poorly controlled DM significantly increases the risk of dementia [6], aggressive management of blood sugar, in turn, heightens the risk of hypoglycemia, which could have disastrous consequences.

In the past decade, diabetic management protocols and healthcare practitioners have primarily focused on optimal glycemic control to prevent hyperglycemia and associated complications in DM patients [7]. However, such singular emphasis has proportionally increased the number of patients reporting severe hypoglycemia. Data suggest that up to 58 to 64% of patients treated with insulin and non-insulin therapies require medical assistance for the management of hypoglycemia over a 6-12 month period [8]. While hypoglycemia is easily treatable and a transient complication, it is not without other short and long-term adverse effects. Hypoglycemia has been shown to increase the risk of micro and macrovascular complications of DM along with the increased risk of falls and fractures [9]. Research also suggests a heightened risk of cardiovascular and all-cause mortality due to hypoglycemia in diabetics [10].

Severe hypoglycemia can also impact brain functions and result in impaired cognitive function and also dementia [11]. Mild cognitive impairment and dementia constitute a spectrum of neurological disorders wherein mild cognitive impairment results in modest impairment in one or more cognitive domains with preserved functional abilities; while dementia is diagnosed when the cognitive impairment is severe enough to compromise social and/or occupational functioning [12]. To date, the association between hypoglycemic events and the risk of dementia has been explored by a few systematic reviews. However, these could include only a limited number of studies and not comprehensively explore the association between the two entities. Given the publication of recent studies, there is a need for updated evidence. Hence, the current systematic review was designed to assess if prior hypoglycemic events increase the risk of dementia in DM patients.

## Materials and methods

We prospectively registered the protocol of our review on PROSPERO with registration no CRD42021287921. The reporting guidelines of the PRISMA statement (Preferred Reporting Items for Systematic Reviews and Meta-analyses) were adhered to for the current review [13].

### Literature search

We undertook a systematic and comprehensive search with the help of a medical librarian to explore the electronic databases of PubMed, Embase, ScienceDirect, and CENTRAL. Google Scholar was used to search the gray literature, but only for the first 400 results of the search query. This was done considering the fact Google Scholar produces a large number of results for each search query and only the initial relevant results were considered. Two authors of the review were involved in the database search which was carried out independently. The time limits of the search were set from the inception of every database to 15th November 2021. Only three search terms were used to maximize the results. The search string consisting of “diabetes” AND “hypoglycemia” AND “dementia” was used for all databases. Following the database search, we deduplicated the results. All the remaining studies were analyzed by their titles and abstracts. Articles relevant to the subject of our review were identified and their full texts were extracted. These articles were then examined by two reviewers independently for final inclusion in the review. Any discrepancies in study selection were resolved by consensus. Finally, we also searched the reference list of included studies to look for any other possible inclusions.

### Eligibility criteria

The inclusion criteria of the review were as follows: (1) All types of cohort (prospective and retrospective) studies that were conducted on patients with DM. There was no restriction on the type of DM in the included studies. (2) Studies were to assess the risk of dementia based on past hypoglycemia events (3) Studies were to report a multivariable-adjusted ratio of the risk of dementia with 95% confidence intervals. We did not predefine dementia and hypoglycemia for the review and any definition used by the included studies was accepted.

Exclusion criteria were: (1) Studies comparing data of DM with non-DM patients (2) Studies only on cognitive impairment (3) Studies not reporting adjusted data (4) Studies on patients with gestational DM and patients not clinically diagnosed as DM (5) Non-English language studies (6) cross-sectional studies as they cannot assess the temporal association between hypoglycemia and dementia and 6) Studies reporting duplicate data. If there were two studies with overlapping data, the study with the largest sample size was included.

#### Data extraction and quality assessment

Two authors independently extracted the following data: author details, publication year, study type, study location, the database used, sample size, male gender, smokers, comorbidities diagnosis of hypoglycemia and dementia, the incidence of hypoglycemia and dementia, variables adjusted in the multivariable analysis and follow-up.

The methodological quality of studies was assessed using the Newcastle-Ottawa scale (NOS) [14]. It was conducted by two authors independent of each other. Any disagreements were solved by a discussion. Studies were assessed for selection of study population, comparability, and outcomes, with each domain being awarded a maximum of four, two, and three points respectively. The maximum score which can be awarded was nine. Studies with nine points were considered to have a low risk of bias, seven to eight points were considered to have a moderate risk of bias and those with scores of six and below were with a high risk of bias.

### Statistical analysis

The meta-analysis was performed using “Review Manager” (RevMan, version 5.3; Nordic Cochrane Centre [Cochrane Collaboration], Copenhagen, Denmark; 2014). Multivariable-adjusted ratios were extracted from individual studies and were pooled to calculate the total effect size as hazard ratios (HR) with 95% CI. This was done using the generic inverse variance function of RevMan. All meta-analyses were conducted using the random-effects model.

Heterogeneity was assessed using the I^2^ statistic. I^2^ values of 25–50% represented low, values of 50–75% medium, and more than 75% represented substantial heterogeneity. We assessed publication bias by visual inspection of funnel plots. A sensitivity analysis was carried out to assess the contribution of each study to the pooled estimate by removing one study one at a time and recalculating the pooled effect estimates for the remaining studies. Subgroup analyses were carried out for studies on the Asian/western population, study type, type of DM, adjustment of glycated hemoglobin (HbA1c), gender, and the number of hypoglycemic events.

## Results

### Study details

**Fig. 1:**
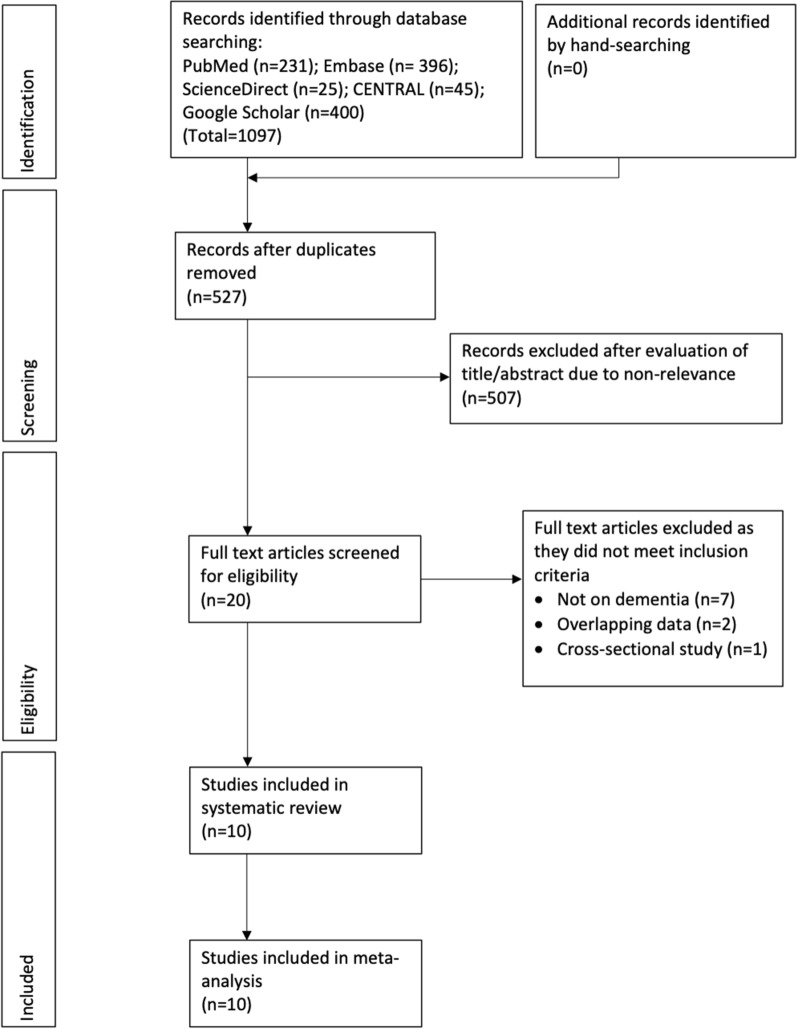
Study flow chart

The PRISMA flow chart of the study is presented in Fig. 1. 1097 articles were found after the literature search. On the exclusion of duplicates, 527 articles remained. 507 of them were excluded after the title and abstract screening and 20 articles were selected for full-text analysis. Ten articles were excluded with reasons and the remaining ten studies [5, 15–23] were included in the systematic review and meta-analysis.

Baseline details of included studies are presented in Table 1. The included studies were published between 2009 and 2021. Four studies were prospective while the remaining were retrospective cohort studies. Only three [15, 18, 20] were conducted in Asian countries (Korea and Taiwan) while all others were from North America or Europe. Data of a total of 1,407,643 patients were analyzed in the included studies. Only one study [17] was conducted on type 1 DM, two studies included a mixed population of both types of DM while the remaining studies included only type 2 DM patients. Most of the studies were registry-based and used international classification of disease (ICD) codes to identify patients with hypoglycemia and dementia. Except for the study of Chin et al. [20] which included all patients with hypoglycemia, all remaining studies included patients only with “severe hypoglycemia” which was very broadly defined as a hypoglycemic event that needed assistance from another person or a visit to a healthcare setup or overnight stay at the hospital. The percentage of patients experiencing hypoglycemia varied from 0.4 to 15.5% in the included studies. The incidence of dementia in the study cohorts ranged from 0.9 to 24.6%. The variables adjusted to assess the risk of dementia due to hypoglycemia varied widely in the included studies. The follow-up of the studies ranged from 1 year to 13.9 years. All studies were of moderate risk of bias and scored 8 on NOS.

**Table 1:** Details of included studies

Study	Location	Database	Study type	Study population	Sample size	Male gender (%)	Diagnosis of hypoglycemia	Diagnosis of dementia	Incidence of hypoglycemia (%)	Incidence of dementia (%)	Adjusted variables	Follow–up	NOS score
Zheng 2021 [16]	UK	U.K. Clinical Practice Research Datalink (1987–2018)	RC	Patient ≥50 years with type 2 DM	457,902	52.1	Using medical codes	Using ICD codes or dementia drug prescription	0.4	6.3	Age, sex, calendar year, region, IMD, smoking status, BMI category, history of comorbidities, DM duration, prescriptions of antidiabetes drugs and baseline HbA1c level	6 years	8
Whitmer 2021 [17]	USA	Kaiser Permanente Northern California Diabetes Registry (1996–2013)	RC	Patient ≥50 years with type 1 DM	2821	52.1	Using ICD codes	Using ICD codes	14.1	5.4	Age, race/ethnicity, HbA1c, depression, nephropathy, and stroke	6.9 years	8
Li 2021 [18]	Taiwan	Taiwan’s National Health Insurance Research Database (2002–2003)	RC	Patient with type 2 DM	677,618	49.1	Using ICD codes	Using ICD codes	5.3	3.8	Age, gender, urbanization level, income–based insurance premium, annual ambulatory visits, township family–income tertiles, and comorbidities (cerebrovascular disease, cardiovascular disease, hypertension, hyperlipidemia, microvascular disease, peripheral neuropathy, depression, head trauma, and end–stage renal disease)	3 years	8
Kim 2020 [15]	Korea	Korean National Health Insurance Service (2002–2015)	RC	Older patients with type 2 DM	11,932	38.1	Using ICD codes	Using ICD codes	50%^#^	24.6	Propensity score matching	1591 days	8
Lee 2019 [19]	USA	Atherosclerosis Risk in Communities study (1996–2013)	PC	Patient with type 2 DM	1263	NR	Using ICD codes	Using ICD codes	15.5	NR	Age, sex, race–centre, education, any APOE ε4 alleles, DM duration, DM medication and fructosamine concentration, systolic blood pressure, use of antihypertensive medication, albuminuria and eGFR	13.9 years	8
Cukierman–Yaffe 2018 [21]	Canada	Outcome Reduction with Initial Glargine Intervention trial (2003–2005)	PC	Patient ≥50 years with type 2 DM and additional cardiovascular risk factors	11,495	67.2	Hypoglycemia requiring the assistance of another person with prompt recovery after oral carbohydrate, intravenous glucose/ glucagon; and/or a documented plasma glucose level of ≤36 mg/dL	Reported dementia or MSME score <24	3.7	0.9	Age, sex, ethnicity, education, prior cardiovascular event, hypertension, depression, smoking, more than two drinks of alcohol per week, an albumin/creatinine ratio ≥30 mg/g, BMI, waist–to–hip ratio, HbA1c, FPG at baseline, glucose– lowering medication use, statin use, ACE/ ARB use, b–blocker use, thiazide use, anti– platelet agent use, lipid profile, blood pressure, serum creatinine, prior DM, and MMSE score at baseline	6.2 years	8
Chin 2016 [20]	Korea	Korea National Diabetes Program (2006–2014)	PC	Patient >60 years with type 2 DM	1957	47	Using ICD codes	Using ICD codes	6.4	2.5	Age, sex, smoking and alcohol status, baseline BMI, diastolic blood pressure, DM duration, previous medical history, baseline medications, total cholesterol, low density cholesterol, and HbA1c	3.4 years	8
Haroon 2015 [22]	Canada	Provincial health data (1995–2007)	RC	Patients with newly diagnosed DM	225,205	49.2	Using ICD codes	Using ICD codes	NR	19.1	Age, sex, income, ethnicity, recent immigration, baseline cardiovascular disease, or kidney disease	7.2 years	8
Yaffe 2013 [5]	USA	Health ABC Study	PC	Patient >70 years with DM	783	52.4	Hypoglycemic event with. Overnight hospital stay	Using ICD codes	7.8	18.9	Age, APOE ε4 status, baseline MSME score, sex, educational level, insulin use, race/ethnicity, HbA1c, myocardial infarction, stroke, and hypertension	1 year	8
Whitmer 2009 [23]	USA	Kaiser Permanente Northern California Diabetes Registry (1980–2007)	RC	Patient ≥55 years with type 2 DM	16,667	54.6	Using ICD codes	Using ICD codes	8.8	11	Age, BMI, race/ethnicity, education, sex, duration of DM, comorbidities, HbA1c, DM treatment, years of insulin use	4.8 years	8

*IMD* index of multiple deprivation, *DM* Diabetes mellitus, *HBA1c* glycated hemoglobin, *BMI* body mass index, *MSME* Mini-Mental State Examination, *ICD* International classification of diseases, *NR* not reported, *eGFR* estimated glomerular filtration rate, *RC* retrospective cohort, *PC* prospective cohort

^#^Does not represent the true incidence of hypoglycemia in the study cohort as 5966 patients with hypoglycemia were matched with another 5966 patients without hypoglycemia for the analysis

### Meta-analysis

**Fig. 2:**
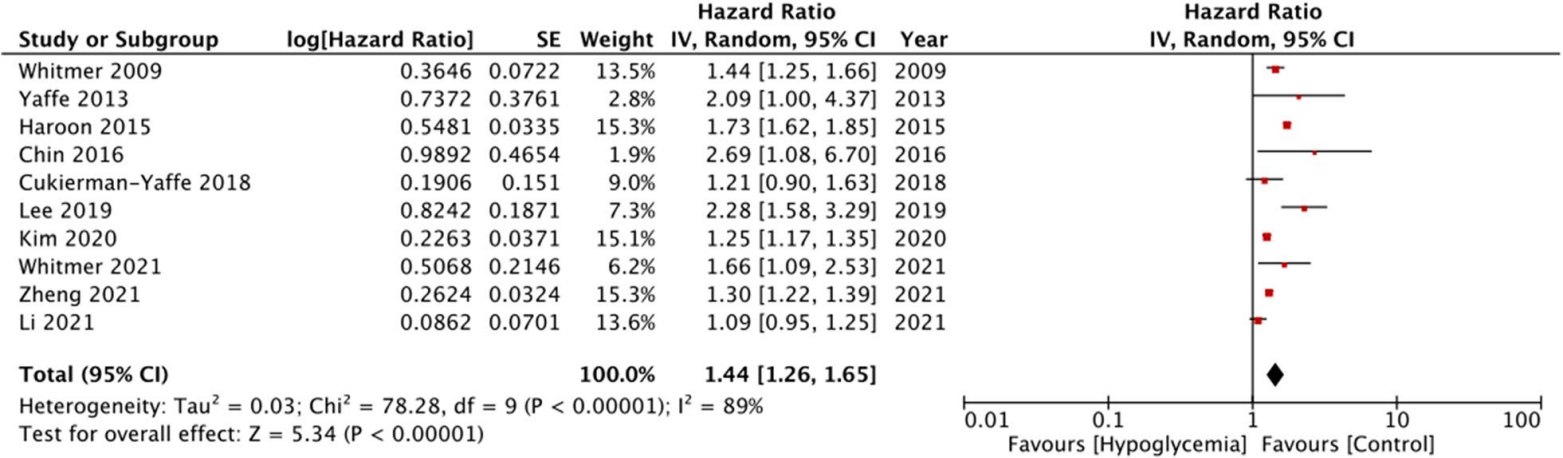
Meta-analysis of the association between hypoglycemic episodes and risk of dementia in DM patients

**Fig. 3:**
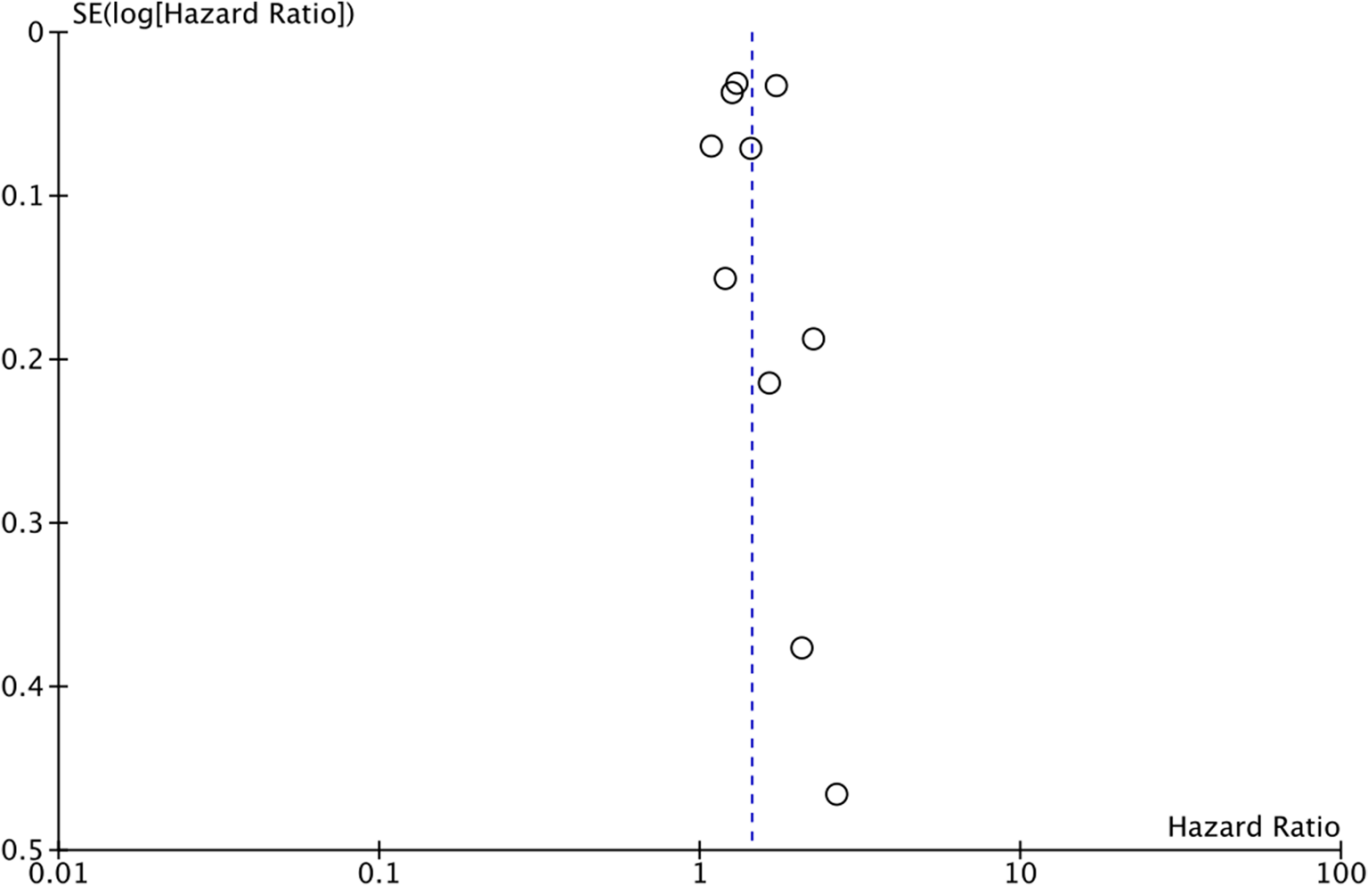
Funnel plot for the meta-analysis of the association between hypoglycemic episodes and risk of dementia

Pooled analysis of all ten studies indicated that hypoglycemic episodes were associated with a statistically significant increase in the risk of dementia in DM patients as compared to those not experiencing hypoglycemic episodes (HR: 1.44 95% CI: 1.26, 1.65 I^2^ = 89% p < 0.00001) (Fig. 2). Results of the sensitivity analysis are presented in Table 2. There was no change in the significance of the results on the exclusion of any study and the pooled HR ranged from 1.35 to 1.50. There was no evidence of publication bias on visual inspection of the funnel plot (Fig. 3).

**Table 2:** Sensitivity analysis

Excluded study	Hazard ratio
Zheng 2021 [16]	1.49 95% CI: 1.26, 1.76 I^2^ = 89% p < 0.00001
Whitmer 2021 [17]	1.43 95% CI: 1.24, 1.65 I^2^ = 90% p < 0.00001
Li 2021 [18]	1.50 95% CI: 1.31, 1.73 I^2^ = 88% p < 0.00001
Lee 2019 [19]	1.39 95% CI: 1.21, 1.59 I^2^ = 89% p < 0.00001
Kim 2020 [15]	1.49 95% CI: 1.27, 1.75 I^2^ = 88% p < 0.00001
Cukierman–Yaffe 2018 [21]	1.47 95% CI: 1.27, 1.70 I^2^ = 90% p < 0.00001
Chin 2016 [20]	1.43 95% CI: 1.25, 1.63 I^2^ = 90% p < 0.00001
Haroon 2015 [22]	1.35 95% CI: 1.22, 1.49 I^2^ = 66% p < 0.00001
Yaffe 2013 [5]	1.43 95% CI: 1.25, 1.64 I^2^ = 90% p < 0.00001
Whitmer 2009 [23]	1.45 95% CI: 1.25, 1.69 I^2^ = 90% p < 0.00001

*CI* confidence interval

Details of sub-group analysis are presented in Table 3. We noted a consistent effect of hypoglycemia in increasing the risk of dementia in both retrospective and prospective studies. On subgroup analysis based on study location, we noted that the association between hypoglycemia and dementia persisted in studies on Asian and western populations. We also noted increased risk of dementia in studies including only type 2 DM and both types of DM. Similar results were noted for studies adjusting and not adjusting for HbA1c. A few studies also reported data on the risk of dementia based on gender and the number of hypoglycemic episodes. Meta-analysis indicated that the risk of dementia with hypoglycemia did not vary based on gender. Also, the risk was significantly increased with both single and ≥2 episodes of hypoglycemia.

**Table 3:** Subgroup analysis

Variable	Groups	No of studies	Hazard ratio
Study population	Asian Western	3 7	1.21 95% CI: 1.02, 1.43 I^2^ = 67% p = 0.03 1.55 95% CI: 1.31, 1.82 I^2^ = 87% p < 0.00001
Study type	Prospective cohort Retrospective cohort	4 6	1.84 95% CI: 1.20, 2.82 I^2^ = 66% p = 0.005 1.37 95% CI: 1.18, 1.59 I^2^ = 93% p < 0.0001
Type of DM	Only type 2 DM Both types of DM	7 2	1.32 95% CI: 1.19, 1.46 I^2^ = 71% p < 0.00001 1.73 95% CI: 1.62, 1.85 I^2^ = 0% p < 0.00001
Adjustment for glycated hemoglobin	Yes No	7 3	1.50 95% CI: 1.27, 1.77 I^2^ = 86% p < 0.00001 1.35 95% CI: 1.07, 1.71 I^2^ = 86% p = 0.01
Gender	Male Female	2 2	1.18 95% CI: 1.05, 1.33 I^2^ = 0% p = 0.006 1.30 95% CI: 1.19, 1.41 I^2^ = 0% p < 0.00001
No of hypoglycemic episodes	1 episode ≥2 episodes	3 3	1.21 95% CI: 1.11, 1.32 I^2^ = 0% p < 0.0001 1.63 95% CI: 1.10, 1.43 I^2^ = 84% p = 0.02

*DM* diabetes mellitus,* CI* confidence interval

## Discussion

DM patients can develop several micro and macro-vascular complications like retinopathy, neuropathy, nephropathy, and cardiovascular diseases, which add to the morbidity of the illness [24]. Additionally, the brain is another target end-organ in diabetics, with research indicating an increased risk of cognitive dysfunction, dementia, and depression amongst DM vs. non-DM patients [4, 25]. In a recent meta-analysis of 122 prospective studies, Xue et al. [4] have comprehensively shown that DM patients have a 1.25 to 1.91 times increased risk of cognitive impairment and dementia as compared to non-diabetic controls. The authors also noted that the risk of dementia is significantly increased by various other DM indicators like high 2-hour post-prandial blood sugar levels, high HbA1c levels, and low and high levels of fasting plasma insulin. While a majority of these markers indicate poor control of blood sugar levels, in this review, we investigated the role of hypoglycemia, which may result from aggressive management of the disease.

Our meta-analysis of 1.4 million patients provides strong evidence on the association between hypoglycemic events and dementia risk in diabetic patients. Our quantitative analysis demonstrated that patients with hypoglycemic episodes have a 44% increased risk of dementia as compared to those not experiencing the same. It is important to note that majority of this data was derived from patients experiencing “severe hypoglycemic” events wherein the complication was critical enough to require assistance or warrant a visit/ overnight admission to the hospital. Most of the studies, due to their study design, could not classify hypoglycemia based on plasma glucose levels and this is an important drawback of the meta-analysis. It is plausible to suggest that the threshold of “severe hypoglycemia” would have been variable in different individuals and at this point, literature is devoid of a ‘cut-off value’ of plasma glucose levels beyond which there would be an increased risk of dementia. However, the validity of our results is strengthened by the fact that there was no change in the significance of the results on sensitivity analysis, and no study was found to overtly influence the pooled effect size. Even after sequential exclusion of studies, the risk of dementia did not show large variations and ranged from 35 to 50%.

The results of our study are in agreement with prior reviews. In a meta-analysis of five studies, Mattishent et al. [26] in 2016 have reported an increased risk of dementia with prior hypoglycemic events in diabetic patients [Odds ratio (OR): 1.68 95% CI: 1.45, 1.95]. However, one of the included studies was cross-sectional in design, reporting risk of mild cognitive impairment and not dementia [11]. Another review [9] published by the same authors in 2019 also demonstrated a statistically significant increased risk of dementia with prior hypoglycemic events (OR: 1.50 95% CI: 1.29, 1.74). Nonetheless, our study is a significant update from their review as we excluded two studies [27, 28] from the past review (due to overlapping data) and included three new studies which significantly raised the statistical power of our analysis. Additionally, unlike the previous review, we also explored the association between hypoglycemia and dementia via several sub-group analyses. One important reason for the subgroup analyses was the high heterogeneity amongst the included studies, with I^2^ of 89% in our primary analysis. While we noted a positive association between hypoglycemic events and dementia irrespective of the study type and study population, the I^2^ values were still in the higher range, indicating an unknown source of heterogeneity.

Long-term glucose control has been an important factor associated with the incidence of diabetic complications. However, its role in diminishing cognitive function has been unclear. A large randomized control trial [29] exploring the effect of long-term glycemic control (target HbA1c of 6% vs. 7–7.9%) on cognitive outcomes of DM patients has shown that intensive glycemic control (HbA1c <6%) results in larger total brain volume and attenuated gray matter loss. However, the trial failed to demonstrate any statistically significant difference in the rate of clinical cognitive decline based on the level of glycemic control. On the other hand, the recent retrospective cohort study of Zheng et al. [16] has shown that higher or unstable HbA1c levels and the presence of diabetic complications results in increased dementia risk amongst diabetics. Considering the importance of long-term glycemic control, we performed a sub-group analysis of studies based on the adjustment for HbA1c in the multivariate analysis, however, we noted no difference between the study groups. One reason for this could be the small number of studies in the subgroup analysis warranting further research.

There are several mechanisms by which hypoglycemia could increase the risk of dementia in DM patients. Since the primary source of energy for the brain is glucose, repeated episodes of dysglycemia could result in structural changes in the brain like reduction of gray matter volume, neuronal damage, and cortical atrophy [17, 30]. Selective damage to the cerebral cortex and the hippocampus, which is involved with memory and learning, could heighten the risk of cognitive impairment [30]. Research also suggests that diabetics have a heightened risk of neuronal damage from hypoglycemia as compared to non-diabetics probably due to altered glucose metabolism or insulin deficiency [31]. The neuronal damage by hypoglycemia may be further enhanced by alteration of ionic hemostasis, increased production of reactive oxygen species, and amyloid precursor proteins [30, 32]. Considering the multiple adverse effects of hypoglycemia on the brain, it is prudent to understand if the number of hypoglycemic events has a role in the risk of dementia. In our review, we noted that just three studies [15, 20, 23] analyzed the impact of the number of hypoglycemic episodes on the risk of dementia. Analysis of the scarce data failed to demonstrate a linear relationship between the number of hypoglycemic events and the risk of dementia. Nevertheless, there is a need for further studies to explore this important variable.

We acknowledge the several limitations of our review. Firstly, the analysis included data from registry-based observational studies which have an inherent bias. Data entry and record-keeping errors can influence the study outcomes. Also, such observational studies can at best reveal associations without proving causality. Secondly, the lack of standard diagnostic criteria for recognition of hypoglycemia and dementia is a major limitation. Inter-clinician, inter-hospital, and inter-study variability in diagnosis could heavily skew the study outcomes. Thirdly, the variables adjusted in each study were, expectedly, not coherent. It is plausible that potential known and some unknown confounding variables could have affected the outcomes of the studies. Fourthly, the baseline risk of dementia can vary with different anti-diabetic medications [33]. The impact of different anti-diabetic medications on the risk of dementia was not adjusted by all included studies. Fifthly, only a limited number of studies assessed the risk of dementia in type 1 DM. This makes it impossible to evaluate if intrinsic characteristics of the pathophysiology of the disease is associated with greater or lesser risk of dementia. Sixthly, our review was restricted to only dementia and did not include patients classified with milder cognitive disorders, like mild cognitive impairment. Lastly, the included studies were from a limited number of countries which just three studies on the Asian population. This limits the generalizability of our results.

The strengths of our study include the large data pooled for the meta-analysis. Only adjusted data was pooled in our study to avoid the impact of known confounders. The stability of the results on sensitivity and several subgroup analyses add to the credibility of our study. The results of our review have important clinical implications considering the large number of patients who suffer from hypoglycemic episodes every year. We believe, diabetologists should dissuade themselves from target-based glucose-lowering which could result in a one-size-fits-all approach and result in adverse events. Variability of glucose levels arising due to age-related physiologic changes and polypharmacy should be taken into account while prescribing glucose-lowering medications, especially in older patients. Furthermore, there exists a reciprocal association between hypoglycemia and dementia [5], wherein hypoglycemia increases the risk of dementia, and dementia in turn increases the risk of hypoglycemia due to difficulty in managing complex DM treatment regimens. Therefore, every effort should be made to balance the benefit of glucose-lowering with the risk of hypoglycemia and avoid a vicious cycle of hypoglycemia-dementia amongst older diabetic patients.

## Conclusions

Evidence from observational studies suggests that prior hypoglycemic events lead to a 44% increased risk of dementia amongst diabetic patients. Further research should focus on the lower level of plasma glucose which significantly increases the burden of dementia. Studies should also focus on whether the risk of dementia increases with the number of hypoglycemic events.

## Data Availability

The data used to support the findings of this study are available from the corresponding author upon request.

## Abbreviation

DM: Diabetes mellitus

## References

[1] Guariguata L, Whiting DR, Hambleton I, Beagley J, Linnenkamp U, Shaw JE (2014). Global estimates of diabetes prevalence for 2013 and projections for 2035. Diabetes Res Clin Pract.

[2] Zheng Y, Ley SH, Hu FB (2018). Global aetiology and epidemiology of type 2 diabetes mellitus and its complications. Nat Rev Endocrinol.

[3] Gudala K, Bansal D, Schifano F, Bhansali A (2013). Diabetes mellitus and risk of dementia: A meta-analysis of prospective observational studies. J Diabetes Investig.

[4] Xue M, Xu W, Ou YN, Cao XP, Tan MS, Tan L (2019). Diabetes mellitus and risks of cognitive impairment and dementia: A systematic review and meta-analysis of 144 prospective studies. Ageing Res Rev..

[5] Yaffe K, Falvey CM, Hamilton N, Harris TB, Simonsick EM, Strotmeyer ES (2013). Association between hypoglycemia and dementia in a biracial cohort of older adults with diabetes mellitus. JAMA Intern Med.

[6] Jia Y, Liu R, Tang S, Zhang D, Wang Y, Cong L (2021). Associations of the glycaemic control of diabetes with dementia and physical function in rural-dwelling older Chinese adults: a population-based study. Clin Interv Aging.

[7] Blonde L, Aschner P, Bailey C, Ji L, Leiter LA, Matthaei S (2017). Gaps and barriers in the control of blood glucose in people with type 2 diabetes. Diabetes Vasc Dis Res.

[8] Silbert R, Salcido-Montenegro A, Rodriguez-Gutierrez R, Katabi A, McCoy RG (2018). Hypoglycemia Among Patients with Type 2 Diabetes: Epidemiology, Risk Factors, and Prevention Strategies. Curr Diab Rep..

[9] Mattishent K, Loke YK (2021). Meta-analysis: association between hypoglycemia and serious adverse events in older patients treated with glucose-lowering agents. Front Endocrinol..

[10] Amiel SA, Aschner P, Childs B, Cryer PE, de Galan BE, Frier BM (2019). Hypoglycaemia, cardiovascular disease, and mortality in diabetes: epidemiology, pathogenesis, and management. Lancet Diabetes Endocrinol.

[11] Gorska-Ciebiada M, Saryusz-Wolska M, Ciebiada M, Loba J (2014). Mild cognitive impairment and depressive symptoms in elderly patients with diabetes: prevalence, risk factors, and comorbidity. J Diabetes Res..

[12] Hugo J, Ganguli M, Dementia, Impairment C (2014). Epidemiology, diagnosis, and treatment. Clin Geriatr Med.

[13] Page MJ, McKenzie JE, Bossuyt PM, Boutron I, Hoffmann TC, Mulrow CD, The PRISMA (2020). statement: An updated guideline for reporting systematic reviews. Int J Surg..

[14] Wells G, Shea B, O’Connell D, Peterson J, Welch V, Losos M, et al. The Newcastle-Ottawa Scale (NOS) for assessing the quality of nonrandomised studies in meta-analyses. https://doi.org/http://www.ohri.ca/programs/clinical_epidemiology/oxford.asp. Accessed 30 Oct 2020.

[15] Kim YG, Park DG, Moon SY, Jeon JY, Kim HJ, Kim DJ (2020). Hypoglycemia and dementia risk in older patients with type 2 diabetes mellitus: a propensity-score matched analysis of a population-based cohort study. Diabetes Metab J.

[16] Zheng B, Su B, Price G, Tzoulaki I, Ahmadi-Abhari S, Middleton L, Glycemic, Control (2021). Diabetic complications, and risk of dementia in patients with diabetes: results from a large U.K. Cohort study. Diabetes Care.

[17] Whitmer RA, Gilsanz P, Quesenberry CP, Karter AJ, Lacy ME (2021). Association of Type 1 diabetes and hypoglycemic and hyperglycemic events and risk of dementia. Neurology.

[18] Li C-Y, Kuo C-L, Chang Y-H, Lu C-L, Martini S, Hou W-H (2021). Association between trajectory of severe hypoglycemia and dementia in patients with type 2 diabetes: a population-based study. J Epidemiol.

[19] Lee AK, Rawlings AM, Lee CJ, Gross AL, Huang ES, Sharrett AR (2018). Severe hypoglycaemia, mild cognitive impairment, dementia and brain volumes in older adults with type 2 diabetes: the Atherosclerosis Risk in Communities (ARIC) cohort study. Diabetologia.

[20] Chin SO, Rhee SY, Chon S, Baik SH, Park Y, Nam MS (2016). Hypoglycemia is associated with dementia in elderly patients with type 2 diabetes mellitus: An analysis based on the Korea National Diabetes Program Cohort. Diabetes Res Clin Pract.

[21] Cukierman-Yaffe T, Bosch J, Jung H, Punthakee Z, Gerstein HC (2019). Hypoglycemia and incident cognitive dysfunction: a post hoc analysis from the origin trial. Diabetes Care.

[22] Haroon NN, Austin PC, Shah BR, Wu J, Gill SS, Booth GL (2015). Risk of dementia in seniors with newly diagnosed diabetes: a population-based study. Diabetes Care.

[23] Whitmer RA, Karter AJ, Yaffe K, Quesenberry CP, Selby JV (2009). Hypoglycemic episodes and risk of dementia in older patients with type 2 diabetes mellitus. JAMA.

[24] Zheng Y, Ley SH, Hu FB (2018). Global aetiology and epidemiology of type 2 diabetes mellitus and its complications. Nat Rev Endocrinol.

[25] Nouwen A, Winkley K, Twisk J, Lloyd CE, Peyrot M, Ismail K (2010). Type 2 diabetes mellitus as a risk factor for the onset of depression: a systematic review and meta-analysis. Diabetologia.

[26] Mattishent K, Loke YK (2016). Bi-directional interaction between hypoglycaemia and cognitive impairment in elderly patients treated with glucose-lowering agents: a systematic review and meta-analysis. Diabetes Obes Metab.

[27] Lin CH, Sheu WHH (2013). Hypoglycaemic episodes and risk of dementia in diabetes mellitus: 7-year follow-up study. J Intern Med.

[28] Mehta HB, Mehta V, Goodwin JS (2017). Association of hypoglycemia with subsequent dementia in older patients with type 2 diabetes mellitus. J Gerontol A Biol Sci Med Sci.

[29] Launer LJ, Miller ME, Williamson JD, Lazar RM, Gerstein HC, Murray AM (2011). Effects of intensive glucose lowering on brain structure and function in people with type 2 diabetes (ACCORD MIND): a randomised open-label substudy. Lancet Neurol.

[30] Sang WS, Hamby AM, Swanson RA (2007). Hypoglycemia, brain energetics, and hypoglycemic neuronal death. Glia.

[31] Bree AJ, Puente EC, Daphna-Iken D, Fisher SJ (2009). Diabetes increases brain damage caused by severe hypoglycemia. Am J Physiol Endocrinol Metab..

[32] Hardy J (2009). The amyloid hypothesis for Alzheimer’s disease: a critical reappraisal. J Neurochem.

[33] Wium-Andersen IK, Osler M, Jørgensen MB, Rungby J, Wium-Andersen MK (2019). Antidiabetic medication and risk of dementia in patients with type 2 diabetes: a nested case-control study. Eur J Endocrinol.

